# Sheep Quickstep while the Floor Rock and Rolls: Visuomotor Lateralization during Simulated Sea Travel

**DOI:** 10.3390/ani9090700

**Published:** 2019-09-18

**Authors:** Andrew Robins, Gabrielle Berthoux, Eduardo Santurtun, Grisel Navarro, Clive J. C. Phillips

**Affiliations:** 1 Centre for Animal Welfare and Ethics, School of Veterinary Science, University of Queensland, Gatton, Queensland 4343, Australia or gabrielle.berthoux@agrosupdijon.fr (G.B.); esanturtun@gmail.com (E.S.);; 2 Agrosup, 21000 Dijon, France; 3 Facultad de Medicina Veterinaria y Zootecnia, Universidad Nacional Autónoma de México, México City 04510, Mexico; 4 Departamento de Medicina Veterinaria, Universidad Católica de Temuco, Temuco 4780000, Chile

**Keywords:** behavior, laterality, locomotion, motion, sheep, transport

## Abstract

**Simple Summary:**

During livestock transport the floor of the vehicle moves in a way that can disturb their balance. This can stress the animals, producing signals that are processed by the right half of their brain. This half of the brain controls movement of the sheep on the opposite, left side of their body. Hence we investigated whether limb movement was more pronounced on this side, providing evidence of stress responses. We found that sheep limb movements were increased in their left hindlimb and right forelimb during balance correction when the floor movements were most unpredictable. This may be explained by sheep using their back right leg as a pivot. We further tested which side sheep lie down on from internet pictures and found a preference for left side lying. We conclude that sheep balance correction shows evidence of a preferred use of limbs, which suggests that sheep are stressed by floor motion.

**Abstract:**

Unpredictable floor motions during transport disturbs animals’ balance, requiring stepping to move the centre of gravity in the direction of body movement. When repeated regularly, this may be stressful, requiring involvement of the right brain hemisphere, hence we investigated the existence of behavioral laterality in sheep during prolonged floor motions. Six sheep were restrained in pairs on a programmable rocking platform, in which they were unable to turn around. They were exposed to three continuous rocking motion treatments (roll, pitch or both) in a regular or irregular pattern for 1 h periods in a changeover design. Right forelimb and left hindlimb diagonal stepping was more frequent in response to the motion treatment of irregular roll and pitch, which previous research has suggested to be the most stressful from heart rate measurements. An overall strategy to maintain balance appeared to be the use of the right hindlimb as a stabilizer, which was repositioned least often of all limbs until towards the end of the hour of experimental treatment. Of each tested pair, sheep restrained on the left side of the rocking floor stepped significantly often than its partner restrained on the right side, and we postulate the existence of visuomotor lateralization as left restrained sheep were unable to view their partner within the field of view of their left eye. We also investigated which side sheep lie down on, which if left lateralized could explain our observed bipedal diagonal control of sheep balance under stress. From the observation of 412 web-based images of sheep, there was an overall left-sided laterality to their lying, as has been observed in cattle. We conclude that stepping activity in sheep in response to a motion stressor is lateralized, providing evidence that floor motion experienced in transport may induce stress responses.

## 1. Introduction

The term ‘lateralization’ refers to specialized neural processes carried out predominantly within either the left or right sides of the brain. Assessment of lateralized behavior in domestic animals is of increasing importance to improving our understanding of welfare issues as it provides a reliable indication of, and changes to, an individual’s affective state [1,2,3]. Behavioral research has identified associations between patterns of motor responses that are favored to one side of the body, and the dominance of the contralateral side of the brain when responding to specific environmental stressors [1,2,3,4]. Thus, increased use of limbs on one side of the body may reliably indicate an underlying shift in the animal’s affective state, as it attempts to cope with a situation that it finds increasingly stressful [5,6,7]. General reviews of the developing field of animal lateralization are found in [8,9,10].

The currently accepted model of lateralized cognitive processing in vertebrate species presents two generally different although complementary modes of analysis for responding to environmental cues [11]. The right hemisphere of the vertebrate brain is primarily concerned with dealing with real-time concerns with the immediate environment and is specialized for a range of functions, including vigilance against potential physical threats. Related specializations for responding to novel objects or sudden changes in the visual surrounds, in addition to social responses, are also primarily driven by right brain processing. Studies have also found a correlation between right brain specializations and asymmetrical control of the autonomic nervous system: The right side of the brain predominantly controls the sympathetic nervous system responses—those primarily concerned with the functions of fight, flight, freezing and reproductive activities [12,13,14]. For these reasons the right side of the vertebrate brain is commonly referred to as the comparatively more “emotional” side of the brain, and is also referred to as the hemisphere concerned with a “negative affect” or “negative valence” [1,2,3], due to its role in directing responses to avoid pain.

By contrast, the left hemisphere (and side) of the vertebrate brain has been found to be primarily concerned with specialized processing involving long-term memories, and connecting abstract concepts to enable stepwise planning to achieve a comparatively complex goal. Specific details are preferentially attended to by the left hemisphere, in contrast to the tendencies for broad, global aspects of the same stimulus attended to by the right hemisphere. Processes carried out by the left side of the brain are able to override those of the right side of the brain as considered, rules-based responses may dominate spontaneous reactions [11]. The left hemisphere of the vertebrate brain is generally regarded as the more “logical” side, and is also referred to the side concerned with a “positive affect” or “positive valence” [1,2,3], due to its role in directing considered or anticipatory responses to reach rewards such as food.

Due to the crossed-lateral organization of the visual, auditory and somatosensory systems (however not the evolutionarily earlier olfactory and gustatory sensory modalities), the reception of sensory information is processed primarily within the opposite side of the brain. For example, visual processing from the respective eyes of vertebrates is commonly referred to as the left eye/right hemisphere and right eye/left hemisphere systems. Although there are species variations with binocular overlap due to differences between frontally and laterally positioned eyes, and variations also in the proportion of optic fibers that come from either eye to the ipsilateral and contralateral sides of the brain [11], general consistencies in response patterns are found that enable clear generalization of left eye/right brain, and right eye/left brain preferences across vertebrate species. Thus the respective use of the terms “left eye system” (LES) and “right eye system” (RES) are typically used to apply to this general organization of lateralized visual processing in vertebrates. Each side of the brain subsequently also controls motor responses back to the opposite, or receiving, side of the body.

Aside from reflex responses, motor responses display the sum output of continuous neural processing drawn from potentially multiple forms of input. In any individual animal, any particular form of motor bias therefore results from a range of factors (reviewed in [15]). Such factors could include a pre-existing injury and asymmetrical effects from pain input pathways, asymmetries of muscular and/or skeletal development from preferential habit or genetic variation, or the involvement of a range of lateralized cognitive processes required to achieve a given motor task [15]. Indeed the valency model of hemispheric specialization infers that prevalent factors such as a pre-existing arousal state may also modulate limb preference in an individual animal.

As most motor output responses are integrated with sensory input and analysis, behavioral experiments in vertebrates (including humans) may more properly indicate visual, or visuomotor biases rather than true motor biases. The findings from behavioral investigations of motor preferences that involve a visual analysis component must therefore be interpreted with caution [15]. Comparatively few experimental designs have been able to isolate the motor from visuomotor biases in vertebrate models, such as the use of reflex righting responses to assess for hindlimb and forelimb preferences in anuran amphibians [16,17]. Moreover, motor bias in prey species in particular infers a weakness or deficiency to one side that may be exploited by a predator. An evolutionarily stable strategy (ESS) model of lateralized responses in prey species has shown that at sufficiently large group sizes, the benefit to the social group of uniform patterns of laterality outweighs the predation cost [18]. The findings of such models and their subsequent elaborations [19], support an earlier “social facilitation” hypothesis suggesting that lateralized cognitive specializations are more likely to be found in social species because they aid processing speed and efficiency for coordinating large group movements in anti-predator defense [20,21,22,23,24]. The social facilitation hypothesis of lateralized cognition is particularly relevant to the domesticated ungulates (e.g., horses, cattle, reindeer, goats and sheep), as their propensity to aggregate as prey species has been directly attributed to their selection for successful domestication [25,26].

### 1.1. Visual Lateralization in Domestic Livestock

Studies of lateralized visual processing have revealed new insight into the cognitive functions of domestic livestock, which are particularly relevant to welfare measures [1,2,3]. In 1979 the first evidence of lateralized visual processing in a non-human species was reported in domestic chicks [27]. In subsequent research, chicks became a model species for understanding cognitive brain lateralization in vertebrates, and in particular regard to hormonal and ontological aspects of its development and strength of expression (summarized in [11]). Early studies utilized brain tissue ablation and monocular eye patching to reveal differential patterns of processing served by the left and right sides of the brain. Currently, simple observation of the preferred or dominant eye that animals within a population chose repeatedly to orient towards experimental stimuli is sufficient to determine or confirm the existence of lateralized cognitive processing [11]. In one example, domesticated reindeer herds have been found to preferentially and spontaneously circle in an anticlockwise direction when challenged with the stress of mustering [28]. Twenty-seven herds out of 30 with between 90 and 200 domestic reindeer exhibited this preference, not otherwise found in smaller herds of less than 20 to 25 individuals [28]. The authors were unable to determine whether the behavioral lateralization was in response to visual or motor lateralization, or a combination of factors. Given that herd size was a critical factor associated with the herd-level lateralization, it would appear that these early data support the social facilitation hypothesis of Rogers [20,21].

Table 1 summarizes significant visual preferences in ungulate species, excluding sheep, responding to a range of specific experimental and environmental stimuli. Domestic sheep have also been assessed for visual preferences to environmentally significant stimuli. While studies similar to those conducted in horses and goats investigating lateralization of visual processing for positive or negative, familiar or unfamiliar human facial expressions have not yet been reported, there is strong evidence of right hemisphere (LES) specialization for such recognition in social conspecifics [29,30]. In the first of a series of studies, sheep were found to have a left visual hemifield (LES) advantage in the identification of conspecific faces, experimentally manipulated ‘hemifaces’, ‘mirrored hemifaces’ and ‘chimeric’ images and that this lateralized effect was strongest with familiar faces [30]. Choice preferences for discrete features most internal or central to the face of socially familiar sheep were most strongly lateralized for the LES [30]. This result was subsequently confirmed as right-hemisphere specializations in electrophysiological and *c-fos* and *zif*/268 mRNA expression changes (summarized in [29]). Together with similar findings from other species, the authors speculate that specializations for facial processing and control of negative emotions might present an efficient way of alleviating stress and anxiety in sheep [29,30].

Repeated detour tests of individual sheep and lambs have been used to determine population level lateralization that support a dominance of the LES for maintaining visual contact with a social flock mate or dam [31]. Trials of individual sheep and unweaned lambs older than three months of age showed an overall preference to repeatedly detour to the right more than the left side of a low barrier to approach another sheep or their dam, although young lambs 4–10 days of age did not show any lateralized preference in the same task [31]. A follow-up study confirmed LES laterality for maintaining visual contact while detouring around a low obstacle in adult sheep, with no overall laterality found in lambs aged 2–3 months age [32]. Furthermore, resumed contact between dams and lambs was found to correlate with significantly increased time spent in close proximity, and greater activity in dams, in sheep found to be lateralized in the detour trials over non-lateralized sheep [32]. Together the findings of the social isolation and facial recognition tests indicate that sheep are lateralized for LES-directed responses in stressful conditions. In a separate experiment, individual sheep trained in a classical conditioning experiment involving a delayed food reward were found to have significantly greater neural activity in the right hemisphere than in the left hemisphere, as determined by functional near-infrared spectroscopy [33]. The authors hypothesized that the difference in activity was associated with a negative affective state, such as frustration with the delay in the anticipated food reward [33].

### 1.2. Motor Lateralization in Domestic Livestock

The limbs of ungulates lack the prehensile carpal and tarsal structures associated with measures of “handedness” as used in primates and other mammals, as well as avian and amphibian species [15]. For this reason, in addition to their bilaterally symmetrical quadrupedal gait, ungulates offer a good contrasting model for understanding the significance of the existence of motor preferences in vertebrates. Spontaneous stepping and recumbent lying behaviors offer ideal motor activities with which to gauge underlying responses to stress, as they are behaviors that are less likely to be influenced by immediate sensory input, such as visually guided reaching and manipulation.

Field studies investigating forelimb preferences in a range of ungulates reveal a trending pattern of motor laterality. Table 2 summarizes forelimb preferences in ungulate species, excluding sheep, scored while performing a range of motor tasks. It is notable from the table that no statistically significant, population-level lateralization for use of the right forelimb has been reported in ungulates across a range of task performances (Table 2).

In trials of adult sheep returning to their flock from an experimentally isolated location, no population level foreleg preference was found for stepping onto an intervening wooden board [31]. In another experiment using pairs of individually crated sheep on a robotic platform programmed to simulate the effects of sea transport, a lateralized effect in the stepping behavior was observed [53]. Specifically, sheep positioned on the left side of each pair were found to step more rapidly and with greater directional variability than its partner on the right side, with attendant differences in heart rate also observed. The authors hypothesized that the sheep positioned on the right were able to monitor their partner directly in the preferred visual hemifield (LES), and thus show a comparatively less elevated response to the stress of irregular motion [53].

The earliest report of motor laterality in a non-human species that we know of was made by Jackson in 1905 (cited in [54]). Jackson reported his observations of cattle lying on their left side in 58.5% of 340 cases, and then 61% of 493 cases, and published his findings in a volume on animal ambidexterity that was not peer-reviewed [54]. While these early observations were statistically significantly different from chance, only later studies confirmed significant left-sided lying preferences in cattle [54,55]. The reported patterns of sidedness in lying behavior appear to vary with a range of environmental factors such as rumen fill, rumination and particularly pregnancy, for which structural asymmetries such as the size and the location of the rumen and its position with respect to the developing neonate may play significant roles (summarized in [55]). It is worthwhile to note that of nine studies published in the scientific literature, none report significant right-sided lying preferences in cattle, while five report significant left-sided lying particularly in pregnant cattle close to term [55]. We are not aware of any similar published studies of lateralized lying preferences in horses or in goats. A study of motor lateralization in day-old lambs found no population level lateralization recumbency lying posture [52], however in the only other report we are aware of a small sample study found that six out of seven ewes preferred to lie down on their left side instead of the right side [56].

The central aim of the two studies reported here is to determine whether adult sheep possess motor lateralization. In the first experiment, pairs of adult sheep were trained to experience being placed alongside each other in individual crates, on a platform that was programmed to simulate movement during sea travel [53]. Movement trials were conducted indoors with the experimenter operating the platform remotely from outside the testing room, to minimize visual bias. The room was windowless and sound attenuated, well-lit, thermostatically controlled and with no obvious visual distractors to influence the behavior of the sheep. Gross motor behaviors and social interactions between the crated sheep, and heart rate measurements, have already been reported [57]. In this study the video-recorded experimental trials are reassessed specifically to score the pattern of limb movements in the sheep to assess for the presence of lateralized motor preferences. The second experiment consisted of a desktop survey of publicly available images of sheep from online sources, to assess for bias in lying behavior.

## 2. Materials and Methods

### 2.1. Experiment 1. The Effects of Floor Motion on Lateralization of Sheep Stepping Responses

Ethical approval was provided by the University of Queensland Animal Ethics Committee (Approval number: SVS/CAWE/315/12/UQ SVS).

#### 2.1.1. Animal Housing and Management

The design of the methodology for exposing sheep to floor movement, including the programming of the movement platform, heart rate monitoring and video recording of behavior have been described in detail elsewhere [57,58]. In brief, six merino cross wethers of approximately 34 months of age were acquired from the University’s flock, with mean weight (±SEM) 44.2 ± 0.1 kg. The sheep were then shorn over the front half of the body to facilitate heart rate monitor placement. Before and after each trial, sheep were kept in a small paddock with ad libitum water and wheaten chaff, as well as free access to the experimental rooms. During the trials, sheep were restrained in pairs in a crate made with three tubular steel bars (0.87 m wide *1.2 m long * 0.95 m high), bisected by a removable barrier. In this manner the sheep were unable to completely turn around and faced in one direction. The crate and video-recording apparatus were surrounded by a white drop-sheet to reduce the potential of any visual cues from differentially influencing the responses of the two crated sheep. Aluminum bowls and plastic bottles were attached to the outside of the crate. A small external mesh barrier was placed to prevent sheep eating from their companion’s bowl.

#### 2.1.2. Regular and Irregular Roll and Pitch Motions

The motion platform was programmed to move in both regular and irregular sequences for roll (side to side) and pitch (end to end) independently or in combination, using two variables, amplitude and period of the platform movements. An irregular sequence program was constructed from thirty separate amplitude and period values that were randomly selected by the software “Visual Studio 2008” (Visual C++ Express Edition: Microsoft Corporation, Redmond, WA, USA). Regular roll and pitch sequences were programmed as the mean amplitude (4.3°) and period (235 ms) of the irregular roll and pitch sequence. A detailed explanation of the methods to obtain both regular and irregular sequences, including the programming commands, as well as the characteristics of the motion platform used to produce roll and pitch movements independently and in combination, is available in [56]. Essentially, regular patterns of motion repeated in a set sequence provided sheep with the opportunity to anticipate floor movement with experience. Irregular patterns of floor movement minimized anticipatory responses in the sheep, by contrast.

#### 2.1.3. Experimental Protocols

Before the start of each experiment, sheep were habituated to the experimental conditions over a period of 32 d to minimize the confounding effects of other potential stressors preceding and during experimental trials. Potential stressors identified were handling of the sheep, use of a ramp to get them into the crate, drinking from a water bottle, feeding on a pelleted diet and adjustment to a new environment in the research facility, including factors such as heart rate monitoring and the researchers’ presence. The first step involved the reduction of fear of researchers by offering high-quality pellets by hand as a positive reinforcer for the sheep in triads every two hours a day for 10 d. The next stage involved different training procedures, including loading and unloading into the crate using a ramp (8 d), clipping the area of skin where the heart rate monitor electrodes would be placed (10 d), attaching the heart rate monitor (7 d) and 3–4 h inside the research facility for feeding, resting and use of the crate (20 d). The training stopped when there were no obvious fear behaviors and the mean heart rate during training was close to resting heart rate. Sheep were then exposed in pairs to six treatments with two factors: Regular and irregular sequences of pitch, roll, and combined roll and pitch. Each treatment was applied to the sheep in the crate for a 60 min period in a 6 × 6 Latin square with one repetition, lasting 12 consecutive days [57]: See Appendix A Table A1). However for this experiment only four sample periods of 5 min each were analyzed (0–5; 18–23; 36–41 and 55–60 min).

In total, each sheep was exposed to 12 treatment periods, days (Appendix A, Table A1). Sheep experienced treatments in six pairs (1 + 2, 3 + 4, 5 + 6, 1 + 4, 3 + 6 and 2 + 5), with pair effects evaluated statistically. During the trials, sheep had ad libitum access to water and a container with 1.5 kg of lucerne pellets (^®®^ Lockyer Lucerne Products PTY. Ltd., Queensland, Australia).

#### 2.1.4. Behavior and Feed and Water Recording

Sheep behavior was recorded continuously in real time by three video cameras/sheep (Kobi CCD Video Camera, Model K-32HCVF, Ashmore, QLD, Australia) during exposure to treatment. A digital video recorder (Kobi H.266, Model XQ-L 900H, Ashmore, QLD, Australia) was used to record the images, and the video data were then analyzed using a continuous recording of each animal and Cowlog 2.0 behavior software for coding of stepping behaviors [59], recorded as individual events.

### 2.2. Experiment 2. Observations of the Lying Side of Sheep

In order to find if sheep were more likely to lie down on their right or left side, 412 images were analyzed from three search engines with input of the key term “sheep lying down”. The three search engines used were Google (200 images), Bing (200 images) and Unsplash (12 images), all of individual sheep. The analysis involved classifying the lying side of each sheep in the picture as left, right or indistinguishable from a side perspective.

### 2.3. Statistical Analysis

Experiment 1.

Data were analyzed using a multiple-factor ANOVA analysis with repeated measures. The variable studied was the frequency of the stepping carried out by the sheep to maintain balance. We investigated the possible interaction between the stepping variable, the random factor ‘sheep’, and the fixed factors of ‘day’, ‘sequence’, ‘period’, ‘treatment’ and ‘position’ in the crate. In addition to these factors, three interactions were tested: Treatment × sequence, period × treatment and period × sequence. The normality of the residuals was tested using the Anderson–Darling test. Since most of the residuals were not normal (*p* < 0.05), a transformation using the logarithm_10_ was applied to the data set. A second ANOVA was carried out on the modified data. When the frequency of movement was significantly different between treatments (*p* < 0.05), a Fisher’s multiple comparison test was used to define the differences between individual treatments. Results are presented both as stepping diagrams with the mean number of steps per 20 min for significant differences, and as tables of mean values with statistical analysis in Appendix A. Where necessary the data was back-transformed by the inverse of the logarithm_10_ function.

Experiment 2.

Images of sheep lying down were scored according to whether there was an obvious left or right side of lying, and the data analyzed using a χ^2^ test (1 df), against the hypothesis that sheep would not have a sidedness bias in lying posture. Significance was accepted at the α < 0.05 level.

## 3. Results

### 3.1. Experiment 1. The Effects of Motion Stress on Lateralization of Sheep Stepping Responses

**Figure 1:**
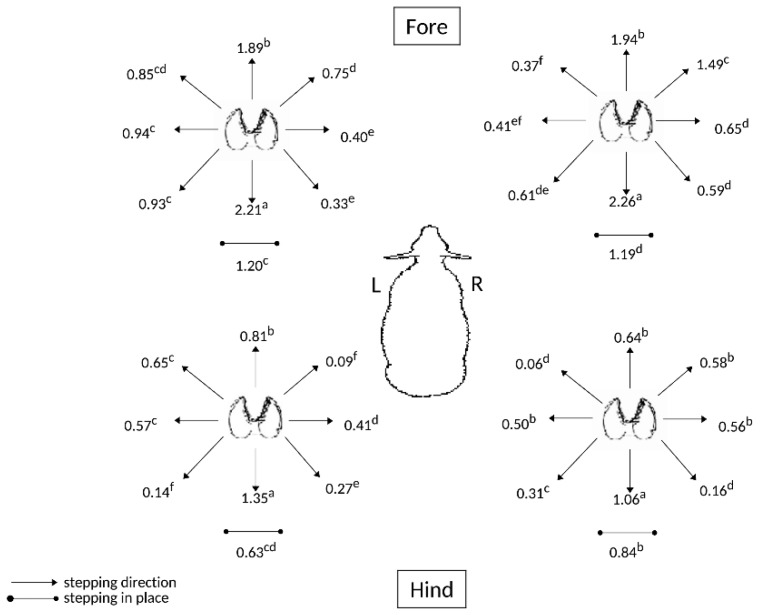
Movement (steps/20 min) of each limb of sheep (*n* = 6) during floor motion, with statistical analysis of differences in the direction of movement within a limb by a Fisher’s comparison test. Different superscript letters (a,b,c,d,e,f) denote significantly different responses (*p* < 0.05) observed for each individual limb.

The means of the stepping data for each limb show that there were some directions that were significantly more favored than others, when used by the sheep to maintain balance during floor motion (Figure 1). The stepping pattern was generally similar for each limb: the most used movement was directly backward, or caudally (denoted with the ‘a’ superscript), then directly forward, or rostrally (‘b’ superscript), then, for movement of the forelimbs and left hindlimbs only, a tendency to step the limbs laterally (‘c’ and ‘d’ superscripts) and comparatively rarely in the medial direction (superscripts ‘d’, ‘e’ and ‘f’). Stepping overall was least frequent in the right hindlimb.

**Figure 2:**
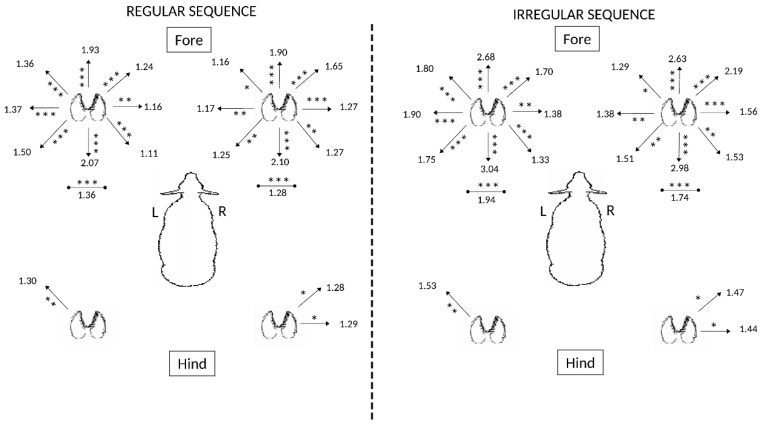
Effects of regularity of movement of the floor on movements (steps/20 min) of the four limbs of sheep (*n* = 6). Directions for significant differences in stepping frequency observed for each respective limb are presented (* *p* < 0.05; ** *p* < 0.02; *** *p* < 0.001).

Figure 2 shows that sheep stepping in response to regular and irregular floor movement predominantly used their forelimbs rather than hindlimbs to adjust to irregular movement (*p* < 0.05). This was notable for both stepping in the rostro-caudal directions and also for stepping in place without translocation of the limb (*p* < 0.001). Hindlimb adjustment was relatively minimal in response to irregular floor movement. Tabulated results are presented in Appendix A, Table A2.

**Figure 3:**
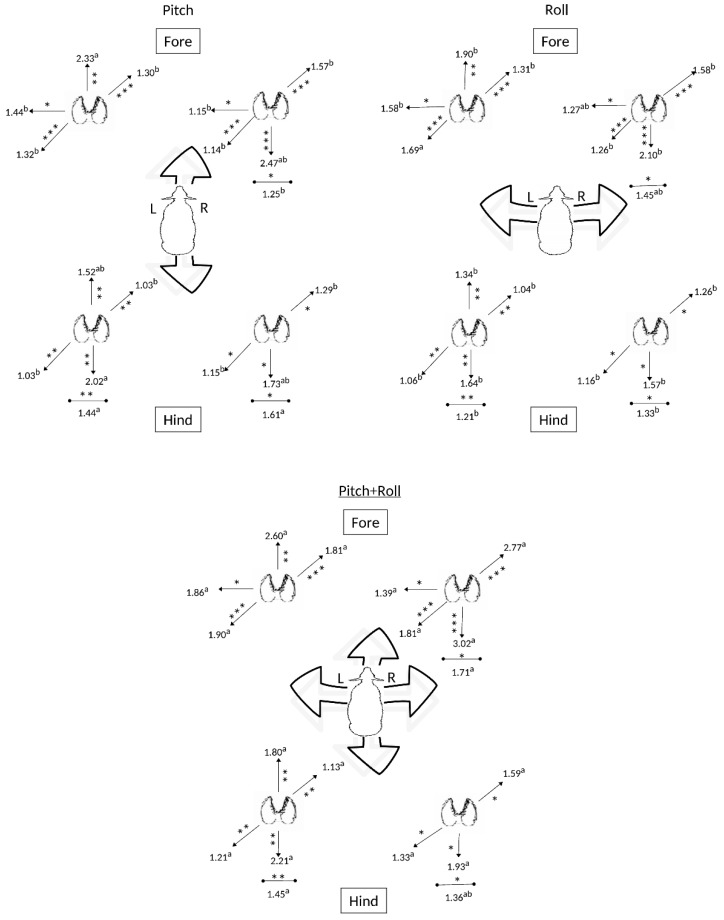
Effects of the type of movement (roll, pitch or the two combined) of the floor on stepping responses (steps/20 min) of the four limbs of sheep (*n* = 6). Directions for which significant differences in stepping frequency are presented (* *p* < 0.05; ** *p* < 0.02; *** *p* < 0.001). Different superscript letters (a,b,c,d,e,f) denote significantly different responses (*p* < 0.05) observed for each individual limb.

Stepping responses to the different types of movement (pitch, roll and the combined roll and pitch floor motion) were greatest for the combined motion (Figure 3). The increased stepping for each individual leg was predominantly on a diagonal orientation from left caudal to right rostral, including stepping within the rostro-caudal direction and, in the forelimbs, also lateral stepping movements to the left (Figure 3). Tabulated results are presented in Appendix A, Table A3.

**Figure 4:**
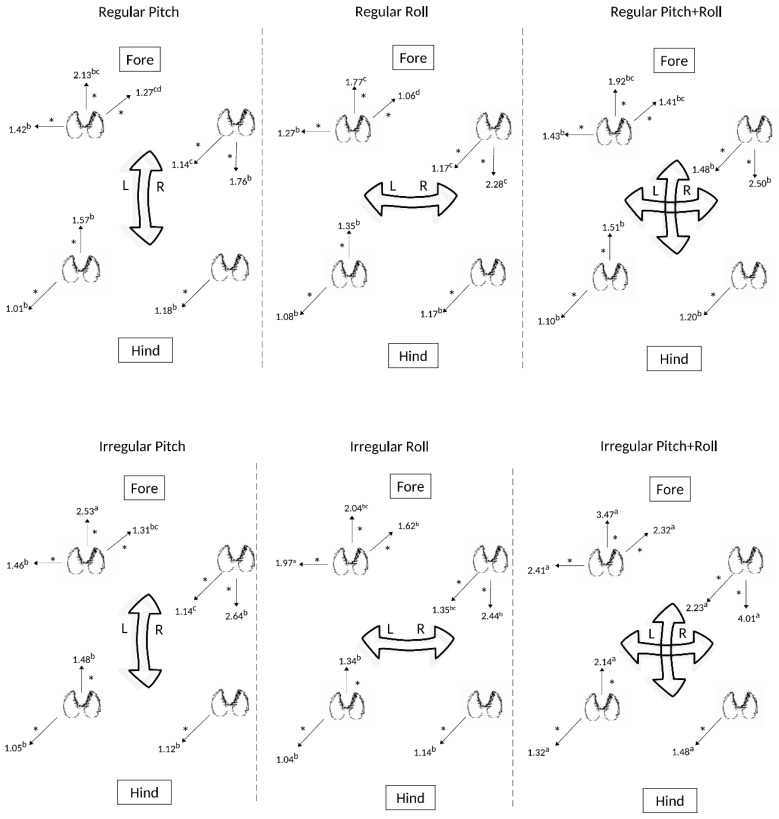
Effects of the type of regular and irregular sequences of floor movement (roll, pitch or the two combined) on stepping responses (steps/20 min) of the four limbs of sheep (*n* = 6). Significantly different frequencies in stepping direction for each respective limb are presented (* *p* < 0.05; ** *p* < 0.02; *** *p* < 0.001). Different superscript letters (a,b,c,d,e,f) denote significantly different responses (*p* < 0.05) observed for each individual limb.

A similar diagonal orientation of stepping responses from caudal left to rostral right was observed across the six interactions between regularity and motion type, for which the irregular sequence of roll and pitch movements generated the greater number of stepping responses (Figure 4). Both right fore- and hindlimbs responded to treatment with primarily caudal stepping, whereas the stepping responses of the left limbs was characterized by both rostral and caudal stepping. Tabulated results are presented in Appendix A, Table A4.

**Figure 5:**
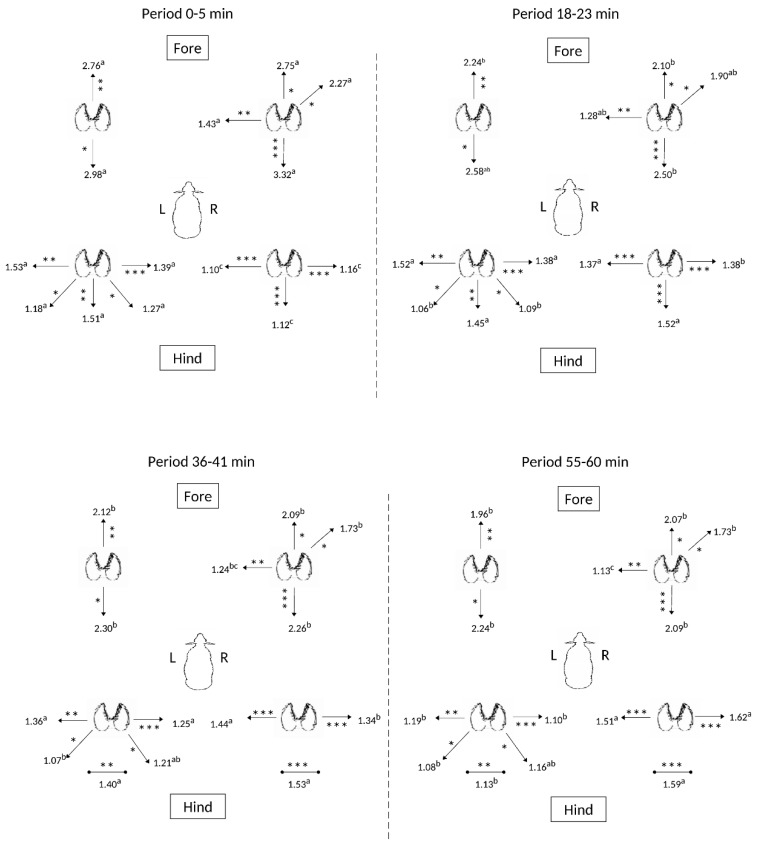
Effects of time within the hour’s exposure on the movements (steps/20 min) of the four limbs of sheep (*n* = 6). Directions for significant differences in stepping frequency observed for each respective limb are presented (* *p* < 0.05; ** *p* < 0.02; *** *p* < 0.001). Different superscript letters (a,b,c,d,e,f) denote significantly different responses (*p* < 0.05) observed for each individual limb.

Figure 5 illustrates that the patterns of stepping responses differed across successive sample intervals over the one hour of floor movement. Both forelimbs and the left hindlimb were used consistently throughout the hour of samples, however there was an increase in observed frequency of stepping with the right hindlimb over the hour and corresponding reduction in use of the left hindlimb for lateral movement in the last period (Figure 5). Moreover, the right forelimb and left hindlimb responded over time in their lateral stepping responses, not the left fore and right hindlimbs (Figure 5), providing further evidence of a caudal left-rostral right diagonal stepping strategy for maintaining balance in response to changing conditions, in this case over time. Tabulated results are presented in Appendix A, Table A5.

**Figure 6:**
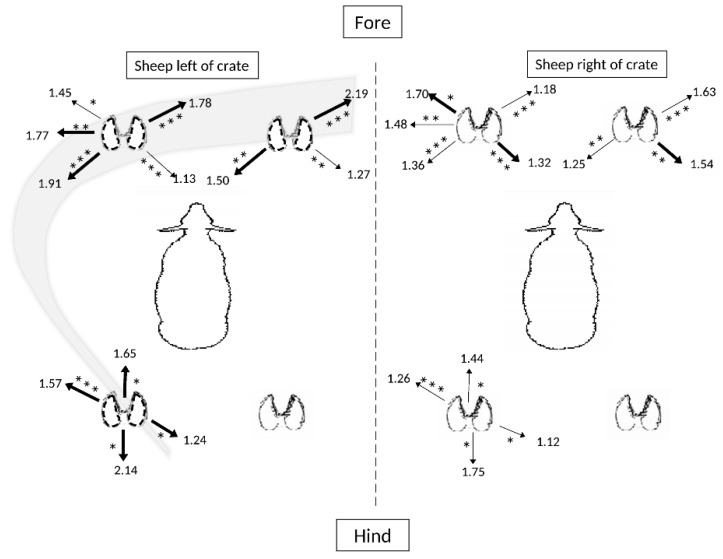
Effects of the side of the crate on movements (step/20 min) of the four limbs of sheep (*n* = 6), with a grey arc indicating the dominant direction of movement. Significantly different frequencies in stepping direction for each respective limb are illustrated (* *p* < 0.05; **p < 0.02; *** *p* < 0.001). Emboldened arrows represent the respectively higher counts of stepping responses for each respective direction.

Comparing sheep on the left or right side of the crate, the sheep on the left side predominantly stepped using their left fore and hindlimbs (Figure 6). More specifically, sheep on the left stepped their forelimbs in a predominantly caudal left-rostral right diagonal, and rarely in the opposite caudal right-rostral left diagonal. In combination the individual stepping responses for sheep on the left, and not the right, tended to have the appearance of pivoting the body in an arc around the fulcrum played by the right hindlimb (grey arc, Figure 6). Tabulated results are presented in Appendix A, Table A6.

Heart rate measurements in the six sheep across the full 60 min of each experimental condition were significantly higher in irregular sequences than for regular floor movement sequences (least mean square values: Regular sequences 81.3 bpm, irregular sequences 83.2 bpm; F-value = 12.51; standard error of the difference between two means (SED) = 0.0188; *p* < 0.001 [57]). For regular sequences, the combination of roll and pitch elicited the slowest mean heart rate (least mean square values: Roll = 83.0^cd^ bpm, Pitch = 86.5^ab^, Roll + Pitch = 77.4^e^ bpm: Least square means with different superscripts were significantly different by the Tukey’s test (*p* < 0.05)). By contrast, the combination of roll and pitch for irregular sequences elicited the highest mean heart rate (least mean square values: Roll = 80.5^d^ bpm, Pitch = 84.7^bc^, Roll + Pitch = 88.3^a^ bpm). The overall interaction was significant (F-value = 50.49; SED = 0.019; *p* < 0.001 [57]).

### 3.2. Experiment 2. Observations of the Lying Side of Sheep

Of the total of 412 sheep lying positions recorded, 45% were lying on their left side, 35% on their right side and in 20% the lying side was unclear (Table 3). Of those images able to be classified, more were lying on the left side (183, 56%) than on the right side (146, 44%), χ_1_^2^ = 4.16, *p* < 0.05). This distribution of more sheep lying on the left than the right side was consistent across all three websites.

## 4. Discussion

This study has demonstrated multiple previously unreported forms of motor lateralization in sheep, and supports a recent report of lateralized limb preferences in a mammal for maintaining balance on a shifting surface [53]. Here we have shown that there appeared to be an underlying preference to use the right hind leg in a central role to essentially anchor the standing posture of the sheep as the other limbs were shifted in position in order to maintain balance. Other preferences were identified—such as a diagonal axis of stepping in a right-rostral to left-caudal orientation, particularly by the forelimbs. However, these preferences might be causally related to the comparatively stable position of the right hindlimb. These experiments were conducted using pairs of sheep crated on a moving platform, and notable differences in positional placement of the sheep were found, indicating a visual affect influencing stepping responses. More particularly, whilst maintaining balance the sheep to the left side appeared to prefer limb movements that would also orient it closer to its social partner (cf. Figure 6). To our understanding this represents visuomotor lateralization in sheep, and confirms the pattern reported earlier by [53]. These findings will be discussed below in terms of the hemispheric valency model. The findings of a desktop survey of images acquired online also identified a dominant left-sidedness for lying in sheep. Together the results have important implications for understanding the welfare requirements of these domesticated animals when coping with environmental stressors.

When coping with floor movement designed to emulate transport motion in a ship, the overall pattern of stepping readjustment to maintain standing balance in sheep pairs was dominated by rostro-caudal, and to a lesser extent lateral, stepping—rather than medial stepping, which presumably would not facilitate standing stability as effectively (cf. Figure 1). These results confirmed the observations of [53]. When subjected to an hour of various treatments involving floor motion to be in a regular or irregular sequence of pitching, rolling and combinations of pitching and rolling, it appeared that the right hindlimb was not significantly involved in translocating from its set standing position, by comparison to the other three limbs (cf. Figure 6). This indicates a particular role for the right hindlimb as a pivot point - a position of strength and stability suggested also by the comparatively moderate incidence of its stepping in place after the first 5-min sample period (cf. Figure 5).

Sheep were observed in this experiment to reposition their forelimbs more often than their hindlimbs. This is likely due to the centre of mass being towards the front of the sheep’s body, because of the weight of the head and length of the neck, requiring relatively more fine positional adjustments of the forelimbs to maintain balance. Forces generated by the forelimbs are generally greater than those exerted by the hindlimbs [60]. Hindlimbs, nevertheless, have a primary role during normal forward movement to deliver the necessary thrust for locomotion, due to their greater size and muscularity [61]. From the relatively lesser number of steps observed in these experiments, the hindlimbs have a primary role, particularly the right hindlimb, for supporting the forelimbs in maintaining balance.

A diagonal stance for balance maintenance is the most common postural adjustment observed in quadrupeds during limb movement [60]. Diagonal stances have also been observed in cats, as this strategy of restricting support forces to a set of two direction-invariant vectors greatly simplifies the problem of maintaining a stance in the face of a force in a horizontal plane; it allows the animals to correct for destabilizing movements of the supporting surface in any direction in the horizontal plane [62]. From the collective data presented in this study, the most parsimonious explanation is that the diagonal axis from the right caudal to left rostral is the preferred direction of postural stability: the left hind and right forelimbs are consequently adjusted in response to shifting postural demands, as reflected in their predominant activity in the left caudal–right rostral plane (cf. Figure 3 and Figure 5). As explained below, this pattern is masked by two key experimental stressors, that of the treatment condition of irregular pitch and roll, and the appearance of cognitive visuomotor lateralization in the left-positioned sheep (cf. Figure 6).

Previous analysis of sheep responses to irregular pitch and roll of the floor have concluded that treatment condition to be the most stressful to the sheep by comparison with the other five combinations of regular or irregular pitch and/or roll treatments [57]. This treatment was the most stressful to sheep as indicated from the number of steps (315/h, compared with the other treatments with 118–208 steps/h: [57]). A similar difference in stepping frequency was observed in our trials, with increased stepping rates in combined pitch and roll (228 steps/h) compared to roll (163 steps/h) and pitch (167 steps/h) alone. Elevated heart rate with reduced variability in response to irregular pitch and roll treatment conditions also indicate this experience to be more aversive than regular floor motion [57]. Whether changes in these physiological measures reflect changes in physiological demand or psychological stress such as frustration [33], or a combination of both factors, remains a matter for speculation. Presumably the greater activity demands in maintaining balance on unpredictable flooring could lead to missed stepping and overbalancing due to fatigue, masking true motor preferences to some degree.

Concerning the effects of crate position, sheep on the left side of the crate stepped more than the sheep on the right side and this finding confirms that outlined in [53]. This suggests that sheep on the left side are comparatively more stressed than the one on the right side, probably because they lack a social companion within their left eye field of view. The preferential stepping pattern apparent for the left-side sheep shown in Figure 6 indicates a drive to turn towards its social partner, perhaps in an effort to monitor her with the LES. No similar pattern of preferences was found for the right-sided sheep as her partner is always located within the LES. Thus, pooling data for left-sided and right-sided sheep may tend to mask the effects of limb placement preferences in motor tasks, due to the difference in their social positions. Sheep stressed by isolation are calmed by the sight of one of their companions [63]. The findings here indicate the importance of the social environment of sheep and reveal lateralized cognitive processing, particularly in stressful contexts. These data of sheep responding in a lateralized manner to environmental stressors correspond with earlier work indicating the existence of lateralized control of a range of hormonal, biochemical and clinical parameters subjected to social separation stress [64].

The lateralized stepping preferences of sheep correspond with the hemispheric valency model raised earlier in the Introduction. The LES—and right side of the brain—is primarily concerned with attending to social and potentially threatening cues, and is strongly linked with sympathetic nervous system control to aid in response to such cues. Here the positional location of the forelimbs and left hindlimb of the sheep without a partner visible to the LES suggests a drive to turn the body to redress the deficit, as also indicated by a corresponding increase in overall stepping behavior, and increased heart rate. By contrast, the left side of the brain (and RES) is primarily concerned with relatively positive, non-threatening cues and recalled strategies. Here the pivotal stabilizing role of the right hindlimb suggests a default function for supporting the upright stance of the sheep, irrespective of the challenges posed by the shifting floor.

The lying preference survey of Experiment 2 indicates another previously unreported form of motor lateralization in sheep, revealing a moderate although significant 56% left-side lying bias in sheep using randomly sampled images. When compared with similar studies of lying preferences in cattle, for whom factors of pregnancy, age and rumen fill are indicated to generally increase the preference for left-sided lying [65,66], it is not currently known how these factors influence the lying behavior in sheep. There may potentially be a direct relationship with left-sided lying and the right hindlimb preference for standing stability found in Experiment 1. Sheep drop to their forelimbs before hindlimbs before lying down, and rise in the reverse sequence by first standing with the hindlimbs. A left-side lying preference would tend to favor the role of the right hind leg as the primary stabilizing limb as the sheep regains the standing position, as suggested from Experiment 1. We are not however aware of any studies that have confirmed a bias for either hind leg for such a function. Other factors may also be at play. For example, all ungulates are prey animals and are known to employ vigilance strategies, such as sleeping while standing. Such animals would be most vulnerable to predation when fully resting, particularly as the approach of predators or startled herd mates may be concealed by foliage, or darkness. The large area of contact between the ground and particularly the lateral side of the body may provide tactile and vibrational information about the distance and direction of walking or running animals around the partially supine individual. In this way, due to the crossed-lateral design of the vertebrate nervous system, the haptic sensory information from the left side of the body can be integrated with the preferential functions of the right brain hemisphere for engaging in anti-predator responses. Similar forms of early warning systems are known to be lateralized in other animals, such as the Mauthner cell reflexes in fish and swimming larval amphibians (reviewed in [8,17]). The existence of such a form of haptic lateralization processing in sheep is however speculative at the time of writing.

## 5. Conclusions

Sheep crated in pairs showed directional lateralization of limb movements in response to floor motion. This bipedal strategy to maintain their equilibrium included using their right forelimb and left hindlimb to create movements in a rostral right and caudal left movement direction, which may provide the most rapid escape strategy in a threatening situation. It is hypothesized that this would enable the left hindlimb to be extracted most rapidly, facilitating rapid escape, in a sheep lying on its left side, which we found to be the dominant side of lying. The right hindlimb potentially acts as a stabilizer, with the forelimbs supporting most of the body weight in the early phase of rising to a standing position. We also suggest that sheep on the left side of the crate showed evidence of more stress than the sheep on the right side, with greater use of their left limbs, the control of which is by the right brain hemisphere, which controls fight or flight responses.

## Figures and Tables

**Table 1:** Population-level visual preferences in ungulate species responding to specific stimuli. LES and RES represent the left and right eye systems, respectively. Visual input from the separate eye systems is processed in the opposite brain hemisphere, each concerned with primarily negative or positive valency modes of analysis (see text).

Species, Task	LES (Right Brain Hemisphere, Negative Valency)	RES (Left Brain Hemisphere, Positive Valency)
Horses, flight in response to approaching human opening and closing an umbrella	LES bias for flight responses [34]	
Horses, inspecting known and unknown humans	LES bias to inspect human [35] *	
Horses, directing agonistic social responses	LES bias [36]	
Wild Przewalski horses, directing agonistic social responses	LES bias [37]	
Wild Przewalski horses, vigilant monitoring during grazing bouts	LES bias [37]	
Horses, approach and inspect a novel red plastic cube	LES bias [38] **	
Horses, simultaneous choice tests of smiling faces		RES bias [39] ***
Horses, electroencephalographic (EEG) recordings taken from alert and quiescent horses during attentional tasks	LES bias [40]	
Cattle, inspecting novel and potentially threatening stimuli	LES bias [41]	
Cattle, approach and inspect non-threatening, novel static objects (balloons and checkerboards) presented bilaterally		RES bias [6]
Cattle, approach and pass an unmasked human		RES bias [42]
Cattle, monitor and permit the approach of an unmasked human		RES bias [43]
Cattle, monitor and permit the approach of a masked human	LES bias [43]	
Cattle, directing agonistic social responses to herd members	No significant LES or RES bias found [44]	
Goats, simultaneous choice tests of smiling faces		RES bias [45] ***

* Consistent preferences irrespective of horses’ previous training to anticipate being approached, saddled and mounted from either the right or left side [35]. ** The strength of lateralization varied according to breed differences and overall emotionality propensity for flight in the horses tested [38]. *** Frowning faces were not preferentially attended by either the LES or RES (horses [39] and goats [45]).

**Table 2:** Forelimb preferences in ungulate species performing specific motor tasks. Note there is no known report of significant right forelimb preferences (left brain hemisphere, positive valency) for population-level motor preferences.

Species (Breed), Task	Left Forelimb (Right Brain Hemisphere, Negative Valency)	No Significant Preferences
Muskoxen, foraging in snow.		no significant bias [46]
Domestic reindeer, foraging in snow.	left forelimb bias [28]	
Horses (thoroughbreds, standardbreds), foreleg bias while grazing.	left forelimb bias [47,48]	
Horses (quarterhorses), foreleg bias while grazing.		no significant bias [48]
Impala, foreleg bias while grazing.	left forelimb bias [49]	
Zebra, foreleg bias while grazing.		weak, non-significant left forelimb bias [49]
Horses (quarterhorses), foreleg bias for stepping off a loading ramp, and also for truck-loading tasks.	left forelimb bias [50] *	
Goats, stepping from elevated platform		no significant bias [51]
Day-old lambs, foreleg bias for initiating walking from standing position.		no population level bias [52] **

* Forelimb preference in the experimental group trended to non-significance during seven successive trials, as did the mean heart rate immediately after loading onto the truck, suggesting a process of habituation [50]. ** In addition, no significant side bias found in tail wagging during suckling [52].

**Table 3:** Number and calculated percentage of sheep lying on their left and right side, or of uncertain laterality, from three online photographic image search engines in Experiment 2.

Weblink	Left-Side Lying Position (% of Total)	Right-Side Lying Position (% of Total)	Unclear Side Position (% of Total)	Total
Google	90 (45)	69 (34)	41 (21)	200
Bing	86 (43)	76 (38)	38 (19)	200
Unsplash	7 (59)	1 (8)	4 (33)	12
Total	183 (45)	146 (35)	83 (20)	412

## Appendix A

**Table A1:** Combinations of sequences and movements tested for each day [57]. The two-factor factorial design for Experiment 1, with motion type (pitch, roll, or both pitch and roll) and treatment sequence (regular or irregular - indicated in normal or italic font, respectively), as the two factors. Individual sheep were designated with the numbers 1 through to 6.

Day	Sheep Pair			Day	Sheep Pair		
	1–2	3–4	5–6		1–4	3–6	2–5
**1**	Roll	*Pitch*	Pitch + Roll	**2**	Pitch	*Pitch + Roll*	*Roll*
**3**	*Pitch*	Pitch	Roll	**4**	*Roll*	Pitch + Roll	*Pitch + Roll*
**5**	Pitch	*Roll*	*Pitch*	**6**	*Pitch + Roll*	Roll	Pitch + Roll
**7**	*Roll*	*Pitch + Roll*	Pitch	**8**	Pitch + Roll	*Pitch*	Roll
**9**	*Pitch + Roll*	Pitch + Roll	*Roll*	**10**	Roll	Pitch	*Pitch*
**11**	Pitch + Roll	Roll	*Pitch + Roll*	**12**	*Pitch*	*Roll*	Pitch

**Table A2:** Effects of regular and irregular patterned sequences of floor movement on the direction of stepping in the four limbs of sheep (*n* = 6). Data presented as (log_10_ + 1) steps/20 min, with back-transformed means provided in brackets. Comparisons with significantly different counts of stepping responses are presented, as also summarized graphically in Figure 2. (L = left and R = Right. SED = standard error of the difference between two means.)

Limb, Step Direction	Sequence Pattern		SED	F-value	*p*-Value
	Regular	Irregular			
Fore L, rostral	0.29 (1.93)	0.42 (2.68)	0.018	13.7	<0.001
Fore L, caudal	0.32 (2.07)	0.48 (3.04)	0.018	20.8	<0.001
Fore L, medial	0.06 (1.16)	0.14 (1.38)	0.011	11.2	0.001
Fore L, lateral	0.14 (1.37)	0.28 (1.90)	0.014	25.9	<0.001
Fore L, step-in-place	0.13 (1.36)	0.29 (1.94)	0.018	20.4	<0.001
Fore L, rostro-lateral	0.13 (1.36)	0.26 (1.80)	0.014	18.2	<0.001
Fore L, rostro-medial	0.09 (1.24)	0.23 (1.70)	0.014	33.4	<0.001
Fore L, caudo-lateral	0.18 (1.50)	0.24 (1.75)	0.015	6.55	0.025
Fore L, caudo-medial	0.05 (1.11)	0.12 (1.33)	0.010	16.2	<0.001
Fore R, rostral	0.28 (1.90)	0.42 (2.63)	0.018	16.3	<0.001
Fore R, caudal	0.32 (2.10)	0.47 (2.98)	0.018	20.7	<0.001
Fore R, medial	0.10 (1.27)	0.19 (1.56)	0.013	13.3	<0.001
Fore R, lateral	0.07 (1.17)	0.14 (1.38)	0.011	12.3	0.001
Fore R, step-in-place	0.09 (1.28)	0.24 (1.74)	0.018	19.3	<0.001
Fore R, rostro-lateral	0.07 (1.16)	0.11 (1.29)	0.011	4.08	0.041
Fore R, rostro-medial	0.22 (1.65)	0.34 (2.19)	0.018	17.3	<0.001
Fore R, caudo-lateral	0.10 (1.25)	0.18 (1.51)	0.013	11.9	0.001
Fore R, caudo-medial	0.10 (1.27)	0.18 (1.53)	0.012	9.36	0.002
Hind L, rostro-lateral	0.11 (1.30)	0.18 (1.53)	0.013	8.46	0.008
Hind R, lateral	0.11 (1.29)	0.16 (1.44)	0.012	4.35	0.043
Hind R, rostro-lateral	0.11 (1.28)	0.17 (1.47)	0.012	5.43	0.018

**Table A3:** Effects of type of floor motion (roll, pitch or the two combined) on stepping behavior of the four limbs of sheep (*n* = 6). Data presented as (log_10_ + 1) steps/20 min, with back-transformed means provided in brackets. Comparisons with significantly different counts of stepping responses are presented. Different superscript letters denote significant differences. These data are also presented graphically in Figure 3 (L = left and R = Right).

Limb, Step Direction	Floor Motion			SED	F-value	*p*-Value
	Pitch	Roll	Pitch + Roll			
Fore L, rostal	0.37 ^a^ (2.33)	0.28 ^b^ (1.90)	0.41 ^a^ (2.60)	0.018	5.67	0.004
Fore L, lateral	0.16 ^b^ (1.44)	0.20 ^b^ (1.58)	0.27 ^a^ (1.86)	0.014	3.81	0.026
Fore L, rostro-medial	0.11 ^b^ (1.30)	0.12 ^b^ (1.31)	0.26 ^a^ (1.81)	0.014	12.5	<0.001
Fore L, caudo-lateral	0.12 ^b^ (1.32)	0.23 ^a^ (1.69)	0.28 ^a^ (1.90)	0.015	13.1	<0.001
Fore R, caudal	0.39 ^b^ (2.47)	0.32 ^b^ (2.10)	0.48 ^a^ (3.02)	0.018	7.97	<0.001
Fore R, medial	0.06 ^b^ (1.15)	0.10 ^ab^ (1.27)	0.14 ^a^ (1.39)	0.011	3.70	0.026
Fore R, step-in-place	0.10 ^b^ (1.25)	0.16 ^ab^ (1.45)	0.23 ^a^ (1.71)	0.018	3.51	0.033
Fore R, rostro-lateral	0.19 ^b^ (1.57)	0.20 ^b^ (1.58)	0.44 ^a^ (2.77)	0.018	24.5	<0.001
Fore R, caudo-medial	0.05 ^b^ (1.14)	0.10 ^b^ (1.26)	0.26 ^a^ (1.81)	0.013	20.4	<0.001
Hind L, rostal	0.18 ^ab^ (1.52)	0.13 ^b^ (1.34)	0.25 ^a^ (1.80)	0.014	6.64	0.002
Hind L, caudal	0.30 ^a^ (2.02)	0.21 ^b^ (1.64)	0.34 ^a^ (2.21)	0.016	6.55	0.002
Hind L, step-in-place	0.16 ^a^ (1.44)	0.08 ^b^ (1.21)	0.16 ^a^ (1.45)	0.014	4.72	0.009
Hind L, rostro-medial	0.01 ^b^ (1.03)	0.02 ^b^ (1.04)	0.05 ^a^ (1.13)	0.005	3.49	0.032
Hind L, caudo-lateral	0.01 ^b^ (1.03)	0.03 ^b^ (1.06)	0.08 ^a^ (1.21)	0.007	7.23	0.001
Hind R, caudal	0.24 ^ab^ (1.73)	0.20 ^b^ (1.57)	0.29 ^a^ (1.93)	0.015	3.10	0.046
Hind R, step-in-place	0.20 ^a^ (1.61)	0.12 ^b^ (1.33)	0.13 ^ab^ (1.36)	0.016	3.01	0.048
Hind R, rostro-lateral	0.11 ^b^ (1.29)	0.10 ^b^ (1.26)	0.20 ^a^ (1.59)	0.012	5.71	0.004
Hind R, caudo-medial	0.06 ^b^ (1.15)	0.06 ^b^ (1.16)	0.12 ^a^ (1.33)	0.009	3.25	0.031

**Table A4:** Effects of regularity and type of motion on the movement patterns of the four limbs of sheep (*n* = 6) subjected to motion treatments and a control. Data presented as (log_10_ + 1) steps/20 min, with back-transformed means provided in brackets. Comparisons with significantly different counts of stepping responses are presented, with these data also presented graphically in Figure 4. Different superscript letters denote significant differences. (L = left and R = Right).

Limb, Step Direction	Regular Sequence, Motion			Irregular Sequence, Motion			Interaction		
	Pitch	Roll	Pitch + Roll	Pitch	Roll	Pitch + Roll	SED	F-value	*p*-Value
Fore L, rostral	0.33 ^c^ (2.13)	0.25 ^c^ (1.77)	0.28 ^bc^ (1.92)	0.40 ^a^ (2.53)	0.31 ^bc^ (2.04)	0.54 ^a^ (3.47)	0.018	3.28	0.039
Fore L, lateral	0.15 ^b^ (1.42)	0.10 ^b^ (1.27)	0.16 ^b^ (1.43)	0.16 ^b^ (1.46)	0.29 ^a^ (1.97)	0.38 ^a^ (2.41)	0.014	4.14	0.017
Fore L, rostro-medial	0.10 ^cd^ (1.27)	0.02 ^d^ (1.06)	0.15 ^bc^ (1.41)	0.12 ^bc^ (1.31)	0.21 ^b^ (1.62)	0.37 ^a^ (2.32)	0.014	4.75	0.016
Fore R, caudal	0.39 ^b^ (1.76)	0.24 ^c^ (2.28)	0.36 ^b^ (2.50)	0.40 ^b^ (2.64)	0.42 ^b^ (2.44)	0.60 ^a^ (4.01)	0.018	3.53	0.030
Fore R, caudo-medial	0.06 ^c^ (1.14)	0.07 ^c^ (1.17)	0.17 ^b^ (1.48)	0.06 ^c^ (1.14)	0.13 ^bc^ (1.35)	0.35 ^a^ (2.23)	0.013	4.59	0.012
Hind L, rostral	0.19 ^b^ (1.57)	0.13 ^b^ (1.35)	0.18 ^b^ (1.51)	0.17 ^b^ (1.48)	0.13 ^b^ (1.34)	0.33 ^a^ (2.14)	0.014	3.60	0.028
Hind L, caudo-lateral	0.03 ^b^ (1.01)	0.03 ^b^ (1.08)	0.04 ^b^ (1.10)	0.02 ^b^ (1.05)	0.02 ^b^ (1.04)	0.12 ^a^ (1.32)	0.007	4.66	0.007
Hind R, caudo-medial	0.07 ^b^ (1.18)	0.07 ^b^ (1.17)	0.08 ^b^ (1.20)	0.05 ^b^ (1.12)	0.06 ^b^ (1.14)	0.17 ^a^ (1.48)	0.009	3.60	0.009

**Table A5:** Effects of time within the hour’s exposure on the stepping responses of the four limbs of sheep (*n* = 6) subjected to motion treatments and a control. Data presented as (log_10_ + 1) steps/20 min, with back-transformed means provided in brackets. Comparisons with significantly different counts of stepping responses are presented, with these data also presented graphically in Figure 5. (L = left and R = Right).

Limb, Step direction	Time Period (Minutes)				SED	F-value	*p*-Value
	0–5	18–23	36–41	55–60			
Fore L rostral	0.44 ^a^ (2.76)	0.35 ^b^ (2.24)	0.33 ^b^ (2.12)	0.29 ^b^ (1.96)	0.018	3.89	0.009
Fore L caudal	0.47 ^a^ (2.98)	0.41 ^ab^ (2.58)	0.36 ^b^ (2.30)	0.35 ^b^ (2.24)	0.018	3.06	0.028
Fore R rostro-lateral	0.44 ^a^ (2.75)	0.32 ^b^ (2.10)	0.32 ^b^ (2.09)	0.32 ^b^ (2.07)	0.018	3.68	0.013
Fore R caudal	0.52 ^a^ (3.32)	0.40 ^b^ (2.50)	0.35 ^b^ (2.26)	0.32 ^b^ (2.09)	0.018	8.50	<0.001
Fore R medial	0.16 ^a^ (1.43)	0.11 ^ab^ (1.28)	0.09 ^bc^ (1.24)	0.06 ^c^ (1.13)	0.011	5.10	0.002
Fore R rostro-lateral	0.36 ^a^ (2.27)	0.28 ^ab^ (1.90)	0.24 ^b^ (1.73)	0.24 ^b^ (1.73)	0.018	4.20	0.01
Hind L medial	0.14 ^a^ (1.39)	0.14 ^a^ (1.38)	0.10 ^a^ (1.25)	0.04 ^b^ (1.10)	0.011	6.33	<0.001
Hind L lateral	0.19 ^a^ (1.53)	0.18 ^a^ (1.52)	0.13 ^a^ (1.36)	0.08 ^b^ (1.19)	0.012	5.48	0.001
Hind L step-in-place	0.18 ^a^ (1.51)	0.16 ^a^ (1.45)	0.15 ^a^ (1.40)	0.05 ^b^ (1.13)	0.014	6.05	0.001
Hind L caudo-lateral	0.07 ^a^ (1.18)	0.03 ^b^ (1.06)	0.03 ^b^ (1.07)	0.03 ^b^ (1.08)	0.007	3.05	0.029
Hind L caudo-medial	0.11 ^a^ (1.27)	0.04 ^b^ (1.09)	0.08 ^b^ (1.21)	0.07 ^ab^ (1.16)	0.009	2.87	0.041
Hind R lateral	0.0 ^c^ (1.16)	0.14 ^b^ (1.38)	0.18 ^b^ (1.34)	0.21 ^a^ (1.62)	0.012	7.55	<0.001
Hind R medial	0.04 ^c^ (1.10)	0.14 ^a^ (1.37)	0.16 ^a^ (1.44)	0.18 ^a^ (1.51)	0.012	8.92	<0.001
Hind R-step-in-place	0.05 ^c^ (1.12)	0.18 ^a^ (1.52)	0.19 ^a^ (1.53)	0.20 ^a^ (1.59)	0.016	8.04	<0.001

**Table A6:** Effects of side positioning of crated sheep on their stepping behavior in response to floor motion (*n* = 6). Data presented as (log_10_ +1) steps/20 min, with back-transformed means provided in brackets. Comparisons with significantly different counts of stepping responses are presented, with these data also presented graphically in Figure 6. (L = left and R = Right).

Limb, Step Direction	Left Crate	Right Crate	SED	F-value	*p*-Value
Fore L, lateral	0.25 (1.77)	0.17 (1.48)	0.014	7.32	0.007
Fore L, rostro-lateral	0.16 (1.45)	0.23 (1.70)	0.014	5.39	0.021
Fore L, rostro-medial	0.25 (1.78)	0.07 (1.18)	0.014	49.9	<0.001
Fore L, caudo-lateral	0.28 (1.91)	0.13 (1.36)	0.015	26.9	<0.001
Fore L, caudo-medial	0.05 (1.13)	0.12 (1.32)	0.010	12.7	<0.001
Fore R, rostro-lateral	0.34 (2.19)	0.21 (1.63)	0.018	16.9	<0.001
Fore R, caudo-medial	0.18 (1.50)	0.10 (1.25)	0.013	10.1	0.002
Fore R, caudo-lateral	0.10 (1.27)	0.19 (1.54)	0.012	10.9	0.001
Hind L, rostral	0.22 (1.65)	0.16 (1.44)	0.014	4.11	0.044
Hind L, caudal	0.33 (2.14)	0.24 (1.75)	0.016	6.71	0.01
Hind L, rostro-lateral	0.20 (1.57)	0.10 (1.26)	0.013	14.9	<0.001
Hind L, caudo-medial	0.09 (1.24)	0.05 (1.12)	0.009	5.61	0.019
